# Single-port versus multi-port totally extraperitoneal (TEP) inguinal hernia repair: A meta-analysis of randomized controlled trials

**DOI:** 10.1097/MD.0000000000030820

**Published:** 2022-11-11

**Authors:** Dimitrios Prassas, Thomas Marten Rolfs, Sascha Vaghiri, Aristodemos Kounnamas, Wolfram Trudo Knoefel, Andreas Krieg

**Affiliations:** a Heinrich-Heine-University and University Hospital Duesseldorf, Department of Surgery, Duesseldorf, Germany; b Catholic Hospital Essen Philippusstift, Teaching Hospital of Duisburg-Essen University, Department of Surgery, Essen, Germany.

**Keywords:** inguinal hernia, meta-analysis, single-access, single-port, TEP, totally extraperitoneal

## Abstract

The value of single-port totally extraperitoneal inguinal hernia repair (STEP) when compared to the conventional multi-port approach (TEP) is still a matter of controversy. We conducted a meta-analysis of randomized controlled trials comparing the feasibility and safety of the above-mentioned techniques.

**Methods:**

A systematic literature search for randomized controlled trials (RCTs) comparing the outcome STEP and TEP in patients with inguinal hernia was conducted. Data regarding postoperative outcomes were extracted and compared by meta-analysis. The Odds Ratio and Standardized Mean Differences with 95% Confidence Intervals (CI) were calculated.

**Results:**

Six RCTs were identified, involving a total of 636 cases (STEP: n = 328, TEP: n = 308). There was a statistically significant difference noted between the 2 groups regarding return to everyday activities favoring the STEP group (SMD = −0.23; 95% CI [−0.41, −0.06]; *P* = .01; 4 studies; *I*^2^ = 9). For the remaining primary and secondary endpoints, intra- and postoperative morbidity, conversion rate, peritoneal tears, major intraoperative bleeding, postoperative haematoseroma, operative time, postoperative pain, chronic pain, cosmetic satisfaction, hernia recurrence and in-hospital length of stay no statistically significant difference was noted between the 2 study groups.

**Conclusions:**

Current evidence suggests that patients who underwent STEP had similar outcomes to the traditional TEP technique with the exception of time to return to everyday activities, which was reported to be shorter in the STEP group.

## 1. Introduction

Inguinal hernia repair is one of the most frequently-conducted surgical operations with more than 200.000 operations being performed annually in Germany alone.^[1]^ In the last few decades, minimally-invasive approaches have gained popularity and have been continuously evolving in order to maximize patient safety, minimize surgical trauma and optimize postoperative outcome. Totally extraperitoneal hernia repair (TEP) repair is one of the most popular procedures with very well documented outcomes since its introduction almost 3 decades ago.^[2,3]^ The single-port totally extraperitoneal hernia repair (STEP) technique emerged as a more refined interpretation of the multi-port TEP approach, following the paradigm of other laparoscopic operations such as single-port Cholecystectomy.^[4]^ However, a consensus with regard to the outcome of STEP when compared to TEP has yet to be reached.

The aim of this study was to perform a meta-analysis of randomized controlled trials (RCTs) comparing the feasibility and safety of standard multi-port TEP and STEP inguinal hernia repair in terms of intra- and postoperative morbidity as well as patient-reported outcomes.

## 2. Materials and methods

This systematic meta-analysis was conducted according to the “Preferred Reporting Items for Systematic Reviews and Meta-analyses” statement.^[5]^

### 2.1. Eligibility criteria

All RCTs comparing the outcome of TEP in cases conducted with single-port and cases with multiple ports were considered for inclusion, regardless of size. To be included in the analysis, studies had to report on at least one of the following outcomes: intraoperative morbidity, perioperative morbidity, conversion rate, peritoneal tears, major intraoperative bleeding, postoperative haematoseroma, operative time, postoperative pain, chronic pain, cosmetic satisfaction, hernia recurrence, time to return to every-day activity and in-hospital length of stay.

### 2.2. Search strategy

A systematic review was independently conducted by two authors (DP and TR) in Scopus and MEDLINE. No language restrictions were applied. The search was limited to publications after the year 2000. Selected papers were screened by both reviewers for eligibility. Discrepancies that arose were resolved by consensus. If needed, a third author (SV) was consulted. The search was performed on April 20, 2022. The combination of the following medical subject headings was used to perform the search: “siltep” or “tep” or “less tep” or “single port” or “single incision” or “single site” or “total extraperitoneal” or “transabdominal preperitoneal” and “miltep” or “conventional tep” or “lap tep” or “total extraperitoneal” and “inguinal hernia repair” or “inguinal herniorrhaphy” or “inguinal hernioplasty”.

### 2.3. Data extraction and outcome measures

A self-designed data extraction form was utilized to independently and blindly extract data of interest in included papers. Primary outcome of interest was intra- and postoperative morbidity. Secondary outcomes of interest included conversion rate, peritoneal tears, major intraoperative bleeding, rate of postoperative haematoseroma, postoperative pain, operative time, hospital stay, delay in return to normal activities, cosmetic satisfaction and recurrence rate. Recorded baseline study characteristics included year of publication, chronic pain, study type, study origin, study duration, sample size, age, gender, body-mass index, number of surgeons involved and surgical skill level, type of implanted mesh, mesh fixation, type of follow-up, and duration of follow-up.

### 2.4. Quality assessment

**Figure 1.**
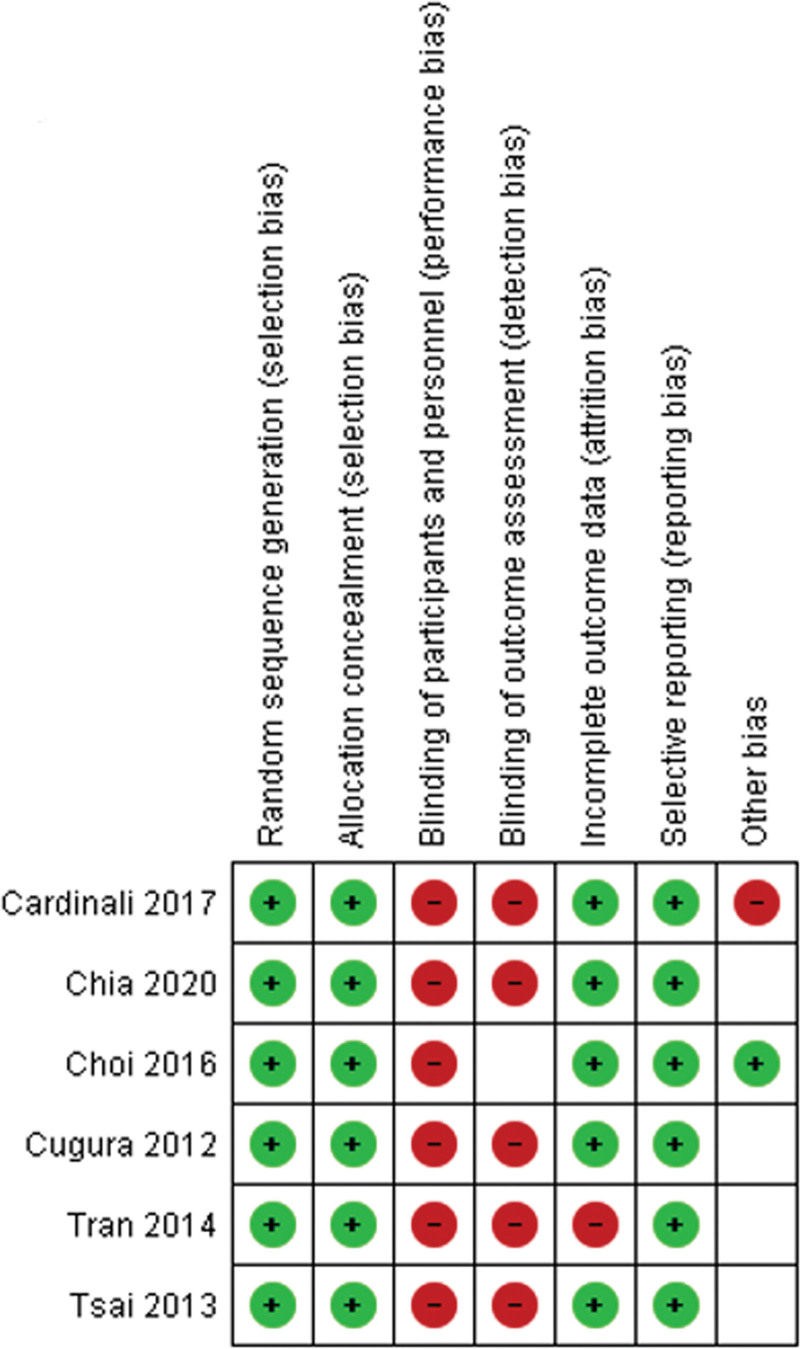
Risk of bias summary.

The risk of bias of included studies was assessed independently by two authors (DP and TR) using the Cochrane Collaboration Risk of Bias tool^[6]^ as part of the data extraction process (Fig. 1). It is a domain-based assessment in which critical evaluations are made separately for 6 different domains of bias or aspects of study design including random sequence generation, allocation concealment, blinding (participants and personnel), blinding of outcome assessment, incomplete outcome data, selective reporting and other forms of bias. The assessors were not blinded to study authors.

The methodological quality of the present meta-analysis was ranked as `high` after implementation of AMSTAR 2 appraisal tool for systematic reviews that include randomized or non-randomized studies of healthcare interventions.^[7]^ The present study was registered in the International Prospective Register of Systematic Reviews (PROSPERO ID: CRD42022327923).

### 2.5. Statistical analysis

Data of interest were analyzed with pairwise meta-analyses. For each outcome of interest summary estimates of treatment effect were calculated with 95% confidence interval (CI). The odds ratio (OR) was chosen as an effect measure for dichotomous endpoints. Standardized mean differences (SMD) were calculated to analyze continuous outcomes. The method described by Hozo et al^[8]^ was implemented to calculate mean and standard deviation in case that only median and range were reported. In case that median and interquartile range were reported, the methods as described by Luo et al^[9]^ and Wan et al^[10]^ were used to calculate mean and standard deviation. The amount of variation by heterogeneity was assessed by the *I*^2^ index. Values exceeding 50% were regarded as markers of substantial heterogeneity. *I*^2^ values above 75% were regarded as markers of high heterogeneity. Summary estimates were calculated with a fixed-effects method in case of low or moderate heterogeneity (*I*^2^ < 50%). All meta-analyses were conducted with the RevMan software (Version 5.3. Copenhagen: The Nordic Cochrane Centre, The Cochrane Collaboration, 2014).

## 3. Results

### 3.1. Study selection and characteristics

**Figure 2.**
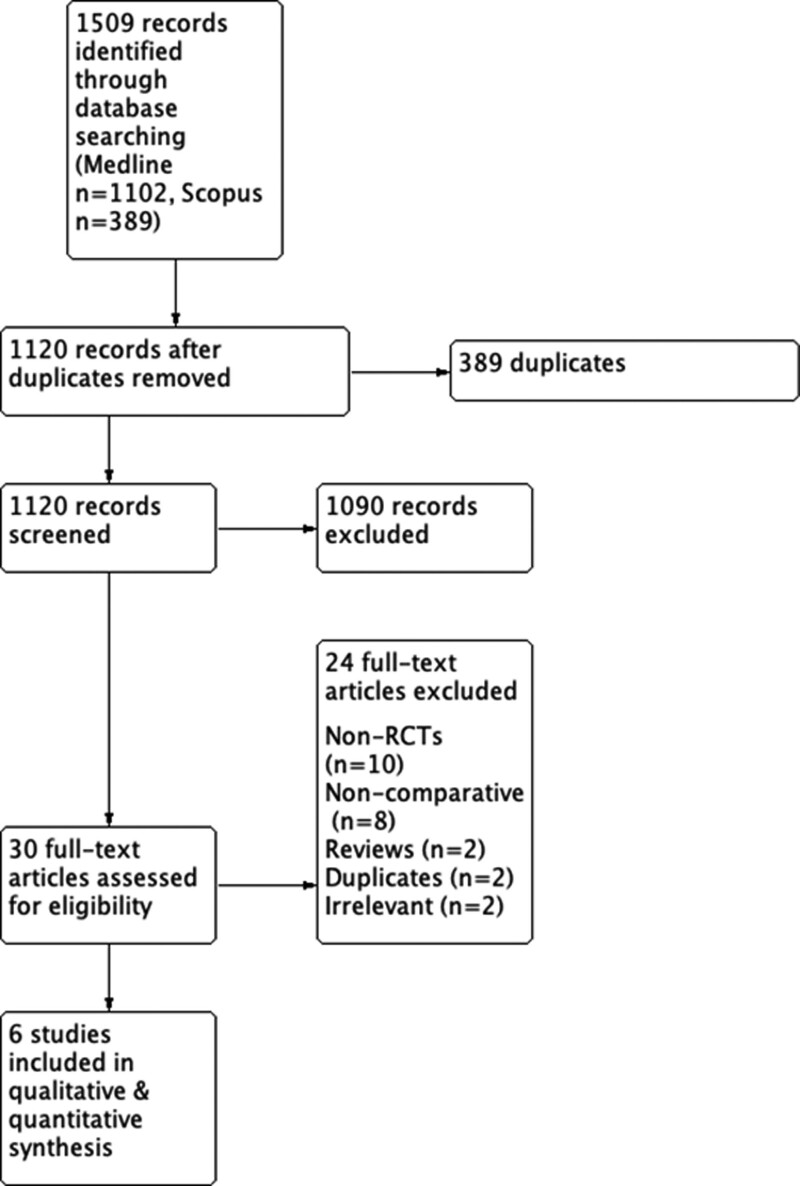
Flow chart of the included studies.

Using our pre-defined literature search strategy and as shown by the PRISMA flow chart, electronic database search identified 1509 studies, including 389 duplicates. Two studies originate from the European^[11,12]^ and 4 from the Australasian region.^[13–16]^ Sample size ranged from 44 participants^[12]^ to 210 participants.^[11]^ Follow-up time ranged from 1 month^[14]^ to 60 months.^[13]^ Two RCTs^[17,18]^ were excluded as the same patient collective was analyzed with longer follow up by Chia et al^[13]^ (Fig. 2). Overall 6 studies were included in the qualitative and quantitative data synthesis, involving a total of 636 cases (STEP: n = 328, TEP: n = 308)^[11–16]^

All included studies were RCTs. Four out of 6 studies stated clearly that hernia incarceration was considered as an exclusion criterion.^[12–15]^ The most commonly implanted prosthetic material was a 10 × 15 cm polypropylene mesh^[11,13,16]^ The inserted mesh was routinely fixated with tacks in 4 studies.^[13–16]^ In one study it was stated that tacks were used only when necessary (40/97 in the STEP patient group and 30/113 in the TEP group).^[11]^ With regard to trocar placement, in 4 out of 6 studies the preferred trocar configuration for multi-port TEP was on the midline^[13–16]^ with the two remaining studies using a triangulated positioning.^[11,12]^ Reported preferred pressures of capnoperitoneum ranged between 8 and 13 mm Hg. Follow-up ranged from 4 weeks to 60 months. Table 1 provides an overview of the study characteristics.

**Table 1:** Study characteristics.

Study	Choi 2016		Cardinali 2017		Tsai 2013		Chia 2020		Cugura 2012		Tran 2014	
Origin	Korea		Belgium		Taiwan		Singapore		Croatia		Australia	
Time period	Jan 2013–Feb 2015		Jan 2013–May 2015		Mar 2010–Dec 2011		N/A		Nov 2008–May 2009		Dec 2011–Feb 2013	
Procedures	TEP	STEP	TEP	STEP	TEP	STEP	TEP	STEP	TEP	STEP	TEP	STEP
Patients	49	50	97	113	50	50	41	42	22	22	49	51
Age	59.5 ± 15.2	57.5 ± 17.2	55 ± 11.5	51.3 ± 11.8	53.2 ± 17.2	55.4 ± 15.1	54.4 ± 15.3	57.3 ± 13.8	51.5 ± 17.9	55.3 ± 18.5	52 ± 36.7	48 ± 16.8
Sex (M/F)	49/0	50/0	91/6	102/11	45/5	44/6	41/0	42/0	22/0	22/0		
BMI (kg/m^2^)	23.1 ± 2.2	23.4 ± 2.9	24.55 ± 4.4	24.85 ± 3.15	24.5 ± 2.8	23.5 ± 3	24.3 ± 3.1	24.4 ± 3.4	N/A		26.1 ± 8.33	25.5 ± 9.76
Number of surgeons	n = 1		n/a		n = 1		N/A		Single surgical team		n = 1	
Surgical skill level	100 TEP, 30 STEP		50 STEP		Experienced in both procedures		N/A		>1000 TEP		>1500 TEP, >300 STEP	
Operative time (min)	61.9 ± 10.7	61.7 ± 11.7	24.55 ± 4.4	24.85 ± 3.15	50.5 ± 39.2	62.5 ± 42.3	51.1 ± 12.5	52.3 ± 16.3	55 ± 11.57	50 ± 23.11	52.5 ± 47.35	48 ± 16.78
Hernia type	40 indirect, 7 direct, 2 pantaloon, 3 scrotal, 1 femoral	44 indirect, 4 direct, 2 pantaloon, 2 scrotal	33 direct, 76 indirect, 5 scrotal, 4 pantaloon	39 direct, 64 indirect, 16 scrotal, 17 pantaloon, 2 femoral	37 indirect, 11 direct, 1 femoral, 1 pantaloon, 2 scrotal	31 indirect, 17 direct, 2 femoral, 4 scrotal	37 direct, 23 indirect, 20 pantaloon	39 direct, 22 indirect, 18 pantaloon, 3 femoral	20 indirect, 2 direct	21 indirect, 1 direct	N/A	N/A
Hernia size	1.49 ± 0.37	1.44 ± 0.39	N/A	N/A	N/A	N/A	2.05 ± 0.55	2.55 ± 0.70	N/A	N/A	Direct left: 3.25 (IR 1), Direct right: 3 (IR 1)	Direct left: 3 (IR 0.5), Direct. Right: 3 (IR = 0.5)
Bilateral hernias (n)	0		29	21	12	17	0		7	3	29	30
Mesh fixation	Tacks, routinely		40/97 tacks	30/113 tacks	Tacks, routinely		Six absorbable tacks, routinely		N/A		Three absorbable tacks, routinely	
Mesh type	13 × 9 cm Parietex^TM^		10 × 15 cm Polypropylene		10 × 15 cm Polypropylene		10 × 15 cm Polypropylene		N/A		11–13 × 15 cm Vyproll™	
Extraperitoneal CO_2_ pressure in mm Hg	8		N/A		13		N/A		N/A		12	
Exclusion criteria	Emergency surgery, ascites, irreducible hernia, previous abd. surgery		Concomitant surgery, inability to tolerate anesthesia		Concomitant surgery, inability to tolerate anesthesia, previous major surgery		Ipsilateral abd. Surgery, emergency surgery, incarcerated hernia		Irreducible hernias, scrotal hernias		Strangulated hernia, unfit for anesthesia, previous extraper. surgery	
Follow Up length in months	1		27 ± 8		6		60		11.25 ± 1.46	11 ± 2	6	
Follow Up rate	100%		100%		50/50	49/50	41/50	42/49	100%		49/49	50/51
Follow Up type	Clinical		Telephone		Clinical		Telephone		N/A		Clinical	

BMI = body-mass index, IR = interquartal range, STEP = single-port totally extraperitoneal hernia repair, TEP = totally extraperitoneal hernia repair.

### 3.2. Study quality and risk of bias

Potential sources of bias are summarized in Figure 1. The main limitations arise from the fact that in surgical intervention trials, the technique used is always evident to the operating surgeon and most of the times evident to the patient as well, rendering blinding of patients and outcome assessors impossible.

### 3.3. Primary outcomes

#### 1.3.3. Intraoperative morbidity.

**Figure 3.**
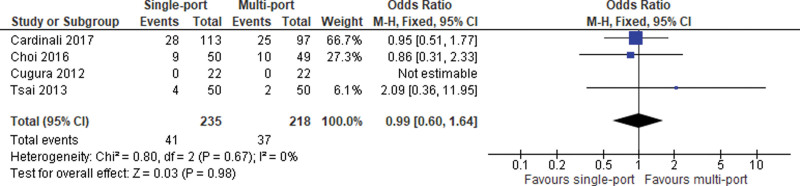
Intraoperative morbidity.

Four included studies reported data on intraoperative complications. No statistically significant difference was noted (OR = 0.99; 95% CI [0.60, 1.64]; *P* = .98; 4 studies; *I*^2^ = 0%; Fig. 3).

#### 2.3.3. Postoperative overall morbidity.

**Figure 4.**
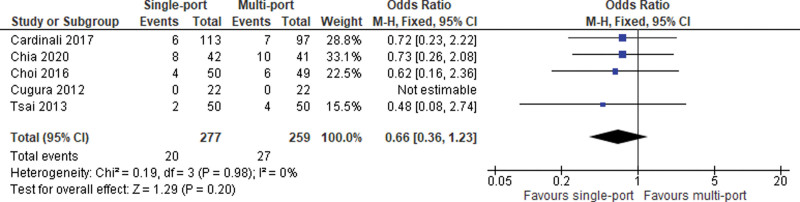
Overall morbidity.

Five studies reported data on postoperative complications. The meta-analysis of pooled data shows no significant difference between the 2 study groups (OR = 0.66; 95% CI [0.36, 1.23]; *P* = .20; 5 studies; *I*^2^ = 0%; Fig. 4).

### 3.4. Secondary outcomes

#### 1.3.4. Major intraoperative bleeding.

**Figure 5.**
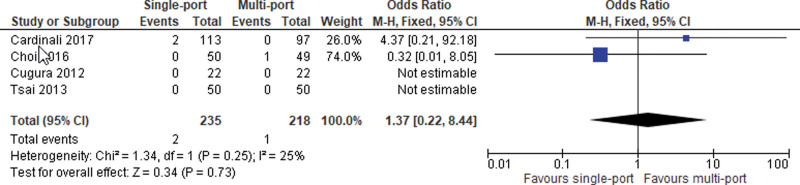
Major intraoperative bleeding.

Data from 4 studies was pooled. No statistically significant difference was detected (OR = 1.37; 95% CI [0.22, 8.44]; *P* = .73; 4 studies; *I*^2^ = 25%; Fig. 5).

#### 2.3.4. Peritoneal tear.

**Figure 6.**
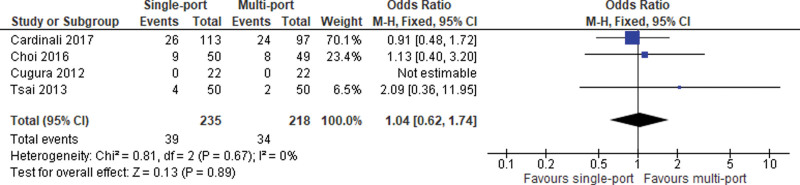
Peritoneal tear.

Four studies reported data on intraoperative tear of the peritoneum and subsequent violation of the peritoneal envelope. Meta-analysis of pooled data reveals a significant no significant difference between the 2 study arms (OR = 1.04; 95% CI [0.62, 1.74]; *P* = .89; 4 studies; *I*^2^ = 0%; Fig. 6).

#### 3.3.4. Conversion rate.

**Figure 7.**
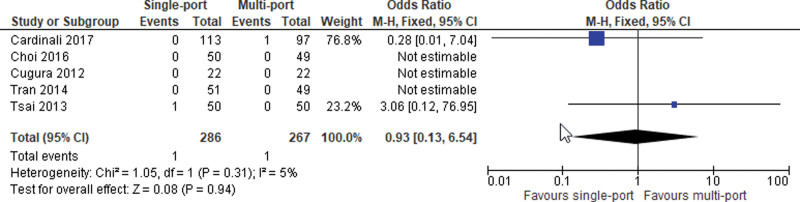
Conversion.

Data from 5 studies was pooled. The meta-analysis failed to show any significant difference of conversion rate between the 2 study arms (OR = 0.93; 95% CI [0.13, 6.54]; *P* = .94; 5 studies; *I*^2^ = 5%; Fig. 7).

#### 4.3.4. Postoperative haematoseroma.

**Figure 8.**
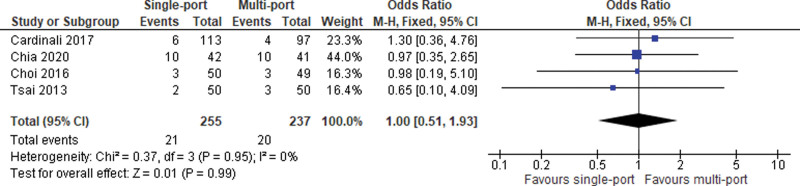
Hematoseroma.

Postoperative incidence of haematoseromas was reported in 4 studies. The meta-analysis of pooled data failed to show any differences between the 2 study groups (OR = 1.00; 95% CI [0.51, 1.93]; *P* = .99; 4 studies; *I*^2^ = 0%; Fig. 8).

#### 5.3.4. Chronic inguinal pain.

**Figure 9.**
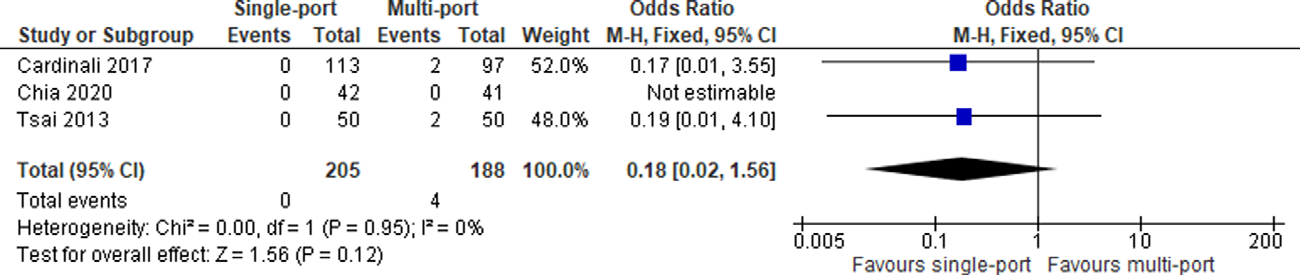
Chronic inguinal pain.

The issue of chronic inguinal pain was addressed in 3 studies. Meta-analysis of these results failed to show any differences between the 2 groups (OR = 0.18 [0.02, 1.56]; 95% CI [0.02, 1.56]; *P* = .12; 4 studies; *I*^2^ = 0%; Fig. 9).

#### 6.3.4. Hernia recurrence.

**Figure 10.**
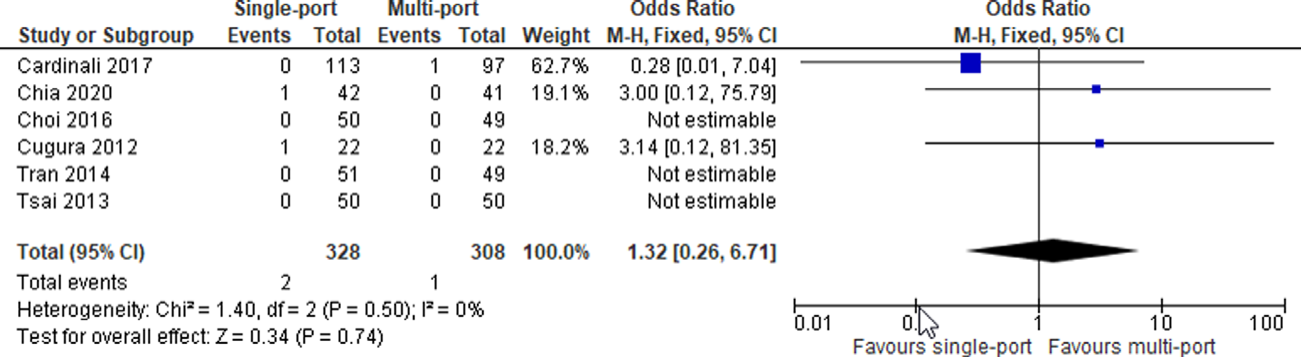
Hernia recurrence.

Data from all studies including a total of 636 cases were pooled. Recurrence rates were not found to be associated with the number of ports used (SMD = 1.32; 95% CI [0.26, 6.71]; *P* = .74; 6 studies; *I*^2^ = 0%; Fig. 10).

#### 7.3.4. Operative time.

Operative time for uni-lateral repair was reported in all included studies and was found to insignificantly differ between the 2 study groups {(SMD = 0.12; 95% CI [-0.13, 0.37]; *P* = .36; 6 studies; *I*^2^ = 58%; Supplemental Digital Content (Fig. S1, http://links.lww.com/MD/H414)}.

#### 8.3.4. Hospital length of stay.

Length of stay was reported in 5 studies. No differences of the length of stay were detectable between the 2 study groups {(SMD = 0.02; 95% CI [−0.15, 0.19]; *P* = .83; 5 studies; *I*^2^ = 0%; Supplemental Digital Content (Fig. S2, http://links.lww.com/MD/H415)}.

#### 9.3.4. Return to everyday activities.

**Figure 11.**
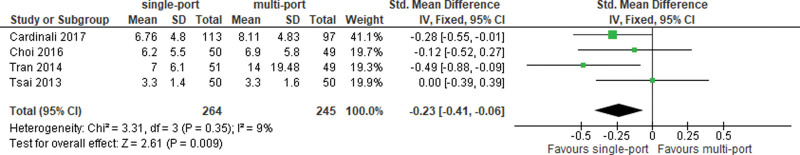
Time to return to normal activity.

Time to return to everyday activities was reported in 4 studies. Meta-analysis revealed a statistically significant difference favoring the STEP group (SMD = −0.23; 95% CI [−0.41, −0.06]; *P* = .01; 4 studies; *I*^2^ = 9%; Fig. 11).

#### 10.3.4. Cosmetic satisfaction

Five studies reported on the cosmetic outcome of the operative technique. Satisfaction scores were higher in all included studies for the STEP subgroup. Three studies concluded that the satisfaction level was significantly higher for the STEP subgroup^[11,13,15]^ whereas the remaining 2 studies did not detect any statistically significant difference regarding that matter.^[14,16]^ Data regarding this outcome could not be pooled due to a high degree of heterogeneity (*I*^2^ = 89%).

#### 11.3.4. Postoperative pain.

Postoperative pain at 24 hours was reported in 5 studies. No statistically significant difference could be revealed between the 2 study groups {(SMD = -0.08; 95% CI [−0.33, 0.16]; *P* = .50; 5 studies; *I*^2^ = 53%; Supplemental Digital Content (Fig. S3, http://links.lww.com/MD/H416)} Sensitivity analysis revealed that after the exclusion of cases from the study of Tran et al^[15]^ where intraroperatively local anesthetic agents were applied, the outcome became more homogenous with a *I*^2^ value of 20%, with the result, however, remaining statistically insignificant.

## 4. Discussion

Minimally invasive techniques for inguinal hernia repair have proven themselves as an efficacious alternative to the traditional open anterior methods with mesh reinforcement in the hands of experienced surgeons, with comparable recurrence rates for both approaches.^[19,20]^ As a result, the focus of herniologists has shifted towards the refinement of already existing methods in order to reach better results in terms of post-operative outcome and socio-economic aspects such as finer cosmesis and shorter time to return to every-day activities. In line with other minimally-invasive operations, the single-port approach or single-incision laparoscopic surgery is being applied on inguinal hernia repair as well. The obvious advantage is a better cosmetic result with the incision becoming practically invisible if created within the umbilical curvature with a further hypothetical advantage being the reduced risk for wound-associated morbidity. On the other hand, single-incision laparoscopic surgery is technically more challenging with different underlying principles to that of traditional multi-port laparoscopy that mainly stem from the loss of triangulation and minimization of the plane of movement.^[21]^ As a result, several trials emerged comparing the single-port with the conventional multi-port operation for inguinal hernia repair that, to our knowledge have been meta-analyzed by Luo et al^[22]^ followed by a revised meta-analysis by Perivoliotis et al^[23]^ 2 years later demonstrating comparable results for both approaches. Since the consensus about whether the STEP approach is advantageous when compared to the multi-port TEP remains controversial, we sought to sched more light upon this issue by conducting an up-to-date meta-analysis of RCTs. The present work included 6 RCTs. Overall incidence of intraoperative morbidity was not statistically different between the 2 study groups. All included studies reported data on intraoperative complications, with the most frequent one being peritoneal tear. Such a lesion becomes clinically relevant when it results to capnoperitoneum that drastically deteriorates the extraperitoneal working space. This can usually occur at various steps of the surgery such as after excessive balloon dilation of the extraperitoneal space, overenergetic preparation of the lateral extraperitoneal space, and hernia-sac reduction. The chances of peritoneal tear become higher in case of preexisting scarring as a result of lower abdominal surgery, a factor that has been shown to generally result to a worse outcome in general, compared to cases with a virgin pre-peritoneal envelope.^[24]^ Cases with previous lower abdominal surgery were excluded in 3 of the meta-analyzed RCTs.^[13–15]^ The second most common intraoperative morbidity reported was major bleeding. In the majority of the cases, it was a result of injury of the inferior epigastric vessels. All those events were dealt with endoscopically and there was no need to convert to open surgery. Overall, 2 conversions were reported, one in the STEP and one in the TEP arm, as a result of clinically relevant peritoneal tear. Overall postoperative morbidity was reported in 5 out of 6 studies and did not reveal any statistically significant differences. There were no statistically significant differences in hospital length of stay. Time to return to everyday activities was reported in 4 studies and was the one and only outcome that was found to differ significantly in favor of the STEP group (SMD = −0.23; 95% CI [−0.41, −0.06]; *P* = .01 with a minimal degree of heterogeneity (*I*^2^ = 9%). Postoperative pain was recorded in 5 studies without showing any statistically significant differences between the 2 study groups. This outcome should be interpreted with particular caution, as it is strongly related to the analgetic regimen of choice. This can vary substantially as a result of different personal preferences and institutional policies. Five studies reported on the cosmetic outcome of the operative technique with satisfaction scores being higher in all included studies for the STEP subgroup. Three studies concluded that the satisfaction level was significantly higher for the STEP subgroup^[11,13,15]^ whereas the remaining 2 studies failed to detect any statistically significant difference. High heterogeneity (*I*^2^ = 89%) did not render pooling of the meta-analyzed data meaningful. This could be attributed to the various follow-up periods recorded, varying from 1 to 60 months postoperatively. This constitutes a major drawback of the included studies and potentially effects various outcomes. Our study has some further limitations. Firstly, the number of RCTs that could be included was not relatively high. A further limitation of our study is the fact that 4 out of 6 trials were conducted in the Australasian region, hence, the included patients may not be representative of general patient population. Surgical expertise of the operating surgeons was variable, and thus a potential source of bias. However, this is to date the first meta-analysis of RCTs focusing on the totally extraperitoneal approach, providing high quality data on the value of STEP compared to the multi-port TEP inguinal hernia repair.

## 5. Conclusion

In conclusion, the present study indicates comparable results between the 2 study groups. Time to return to everyday activities is the only outcome of interest found to differ significantly, favoring the single-port approach. STEP inguinal hernia repair should be offered as an alternative option to the tri-port TEP, if expertise is available, but does not result in a substantially superior outcome compared to the latter. Further high-quality randomized control trials with longer follow-up periods are needed to verify our findings.

## Author contributions

**Conceptualization:** Dimitrios Prassas, Wolfram Trudo Knoefel.

**Data curation:** Dimitrios Prassas, Thomas Marten Rolfs.

**Formal analysis:** Dimitrios Prassas, Thomas Marten Rolfs, Sascha Vaghiri, Aristodemos Kounnamas.

**Investigation:** Dimitrios Prassas, Sascha Vaghiri, Aristodemos Kounnamas.

**Methodology:** Dimitrios Prassas, Thomas Marten Rolfs, Sascha Vaghiri, Wolfram Trudo Knoefel.

**Project administration:** Wolfram Trudo Knoefel, Andreas Krieg.

**Resources:** Thomas Marten Rolfs, Aristodemos Kounnamas, Andreas Krieg.

**Software:** Dimitrios Prassas, Thomas Marten Rolfs, Aristodemos Kounnamas.

**Supervision:** Aristodemos Kounnamas, Andreas Krieg.

**Validation:** Dimitrios Prassas, Sascha Vaghiri, Aristodemos Kounnamas, Andreas Krieg.

**Visualization:** Dimitrios Prassas, Sascha Vaghiri, Wolfram Trudo Knoefel, Andreas Krieg.

**Writing – original draft:** Dimitrios Prassas.

**Writing – review & editing:** Dimitrios Prassas, Sascha Vaghiri, Wolfram Trudo Knoefel, Andreas Krieg.

## Supplementary Material



## References

[1] Jähne J. Surgery of inguinal hernia. Chirurg, 2001;72:456–69. 11357542 10.1007/s001040051331

[2] Dulucq J. Treatment of inguinal hernia by insertion of a subperitoneal patch under pre-peritoneoscopy. Chirurgie. 1992;118:83–5. 1306431

[3] Simons M Aufenacker T Bay-Nielsen M. European Hernia society guidelines on the treatment of inguinal hernia in adult patients. Hernia. 2009;13:343–403. 19636493 10.1007/s10029-009-0529-7 PMC2719730

[4] Podolsky E Rottman S Poblete H. Single port access (SPA) cholecystectomy: a completely transumbilical approach. J Laparoendosc Adv Surg Tech. 2009;19:219–22. 10.1089/lap.2008.0275 19260790

[5] Moher D Liberati A Tetzlaff J. Preferred reporting items for systematic reviews and meta-analyses: the PRISMA statement. PLoS Med. 2009;6:e1000097. 19621072 10.1371/journal.pmed.1000097 PMC2707599

[6] Higgins J Altman D Gøtzsche P. The Cochrane collaboration’s tool for assessing risk of bias in randomised trials. BMJ. 2011;343:d5928. 22008217 10.1136/bmj.d5928 PMC3196245

[7] Shea B Reeves B Wells G. AMSTAR 2: a critical appraisal tool for systematic reviews that include randomised or non-randomised studies of healthcare interventions, or both. BMJ. 2017;21:j4008. 10.1136/bmj.j4008 PMC5833365 28935701

[8] Hozo S Djulbegovic B Hozo I. Estimating the mean and variance from the median, range, and the size of a sample. BMC Med Res Methodol. 2005;20:13. 10.1186/1471-2288-5-13 PMC1097734 15840177

[9] Luo D Wan X Liu J. Optimally estimating the sample mean from the sample size, median, mid-range and/or mid-quartile range. Stat Methods Med Res. 2018;27:1785–805. 27683581 10.1177/0962280216669183

[10] Wan X Wang W Liu J. Estimating the sample mean and standard deviation from the sample size, median, range and/or interquartile range. BMC Med Res Methodol. 2014;14:135. 25524443 10.1186/1471-2288-14-135 PMC4383202

[11] Cardinali L Mazzetti C Cadenas Febres A. Prospective randomized study comparing single-incision laparoscopic versus multi-trocar laparoscopic totally extraperitoneal (TEP) inguinal hernia repair at 2 years. Surg Endosc. 2018;32:3262–72. 29362907 10.1007/s00464-018-6045-z

[12] Cugura J Kirac I Kulis T. Comparison of single incision laparoscopic totally extraperitoneal and laparoscopic totally extraperitoneal inguinal hernia repair: initial experience. J Endourol. 2012;26:63–6. 21999423 10.1089/end.2011.0352

[13] Chia D Lomanto D Wijerathne S. Patient-reported outcomes and long-term results of a randomized controlled trial comparing single-port versus conventional laparoscopic inguinal hernia repair. World J Surg. 2020;44:2191–8. 32123978 10.1007/s00268-020-05443-z

[14] Choi B Jeong W Lee I. Single-port versus conventional three-port laparoscopic totally extraperitoneal inguinal hernia repair: a randomized controlled trial. Hernia. 2016;20:789–95. 27142209 10.1007/s10029-016-1499-1

[15] Tran H Turingan I Tran K. Potential benefits of single-port compared to multiport laparoscopic inguinal herniorraphy: a prospective randomized controlled study. Hernia. 2014;18:731–44. 24824813 10.1007/s10029-014-1261-5

[16] Tsai Y Ho C Tai H. Laparoendoscopic single-site versus conventional laparoscopic total extraperitoneal hernia repair: a prospective randomized clinical trial. Surg Endosc. 2013;27:4684–92. 23949480 10.1007/s00464-013-3116-z

[17] Wijerathne S Agarwal N Ramzy A. A prospective randomized controlled trial to compare single-port endo-laparoscopic surgery versus conventional TEP inguinal hernia repair. Surg Endosc. 2014;28:3053–8. 24902814 10.1007/s00464-014-3578-7

[18] Wijerathne S Agarwal N Ramzi A. Single-port versus conventional laparoscopic total extra-peritoneal inguinal hernia repair: a prospective, randomized, controlled clinical trial. Surg Endosc. 2016;30:1356–63. 26162422 10.1007/s00464-015-4378-4

[19] Eker H Langeveld H Klitsie P. Randomized clinical trial of total extraperitoneal inguinal hernioplasty vs lichtenstein repair: a long-term follow-up study. Arch Surg. 2012;47:256–60. 10.1001/archsurg.2011.2023 22430907

[20] Myers E Browne K Kavanagh D. Laparoscopic (TEP) versus Lichtenstein inguinal hernia repair: a comparison of quality-of-life outcomes. World J Surg. 2010;4:3059–64. 10.1007/s00268-010-0730-y 20703474

[21] Greaves N Nicholson J. Single incision laparoscopic surgery in general surgery: a review. Ann R Coll Surg Engl. 2011;93:437–40. 21929912 10.1308/003588411X590358 PMC3369327

[22] Luo S Wu S Lai H. Single-incision laparoscopic inguinal hernioplasty versus conventional laparoscopic inguinal hernioplasty. 2017. Surg Innov. 2017;24:171–82. 28164741 10.1177/1553350617690308

[23] Perivoliotis K Tzovaras G Sarakatsianou C. Current status of single-port versus multi-port approach in laparoscopic inguinal hernia mesh repair: an up-to-date systematic review and meta-analysis. Hernia. 2019;23:217–33. 30617931 10.1007/s10029-018-01876-7

[24] Prassas D Rolfs T Knoefel W. Meta-analysis of totally extraperitoneal inguinal hernia repair in patients with previous lower abdominal surgery. Br J Surg. 2019;106:817–23. 30912849 10.1002/bjs.11140

