# Supplementary figures and images for: Single-port versus multi-port totally extraperitoneal (TEP) inguinal hernia repair: A meta-analysis of randomized controlled trials
Source: Medicine (Baltimore). 2022 Nov 11;101(45):e30820. doi: 10.1097/MD.0000000000030820 (PMC10662824; doi:10.1097/MD.0000000000030820)

## Supplemental Digital Content (Figure S1). Operative time

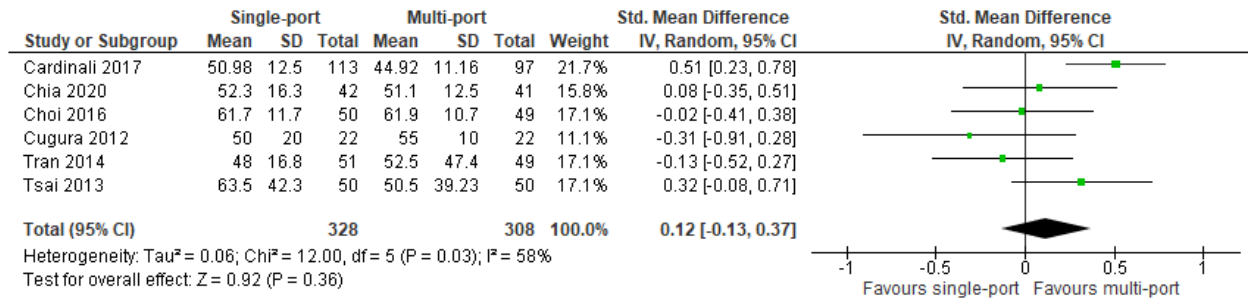

Supplement: Supplementary file 1 [file medi-101-e30820-s001.pdf]

## Supplemental Digital Content (Figure S2). In-hospital length of stay

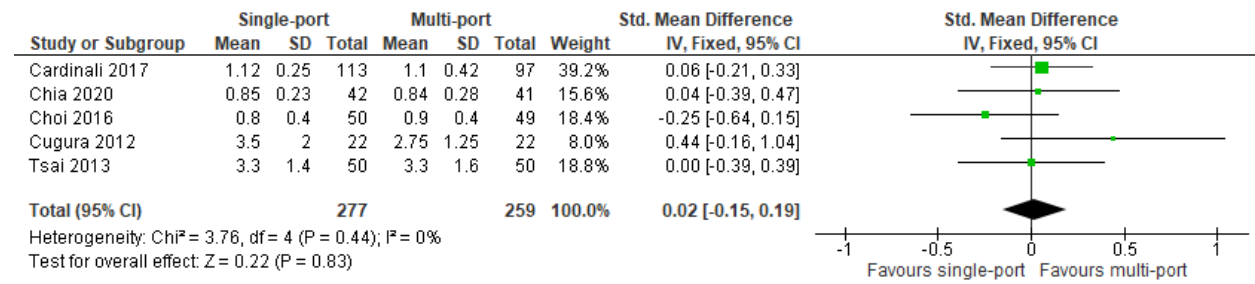

Supplement: Supplementary file 2 [file medi-101-e30820-s002.pdf]

# Supplemental Digital Content (Figure S3). Postoperative pain at 24 h.

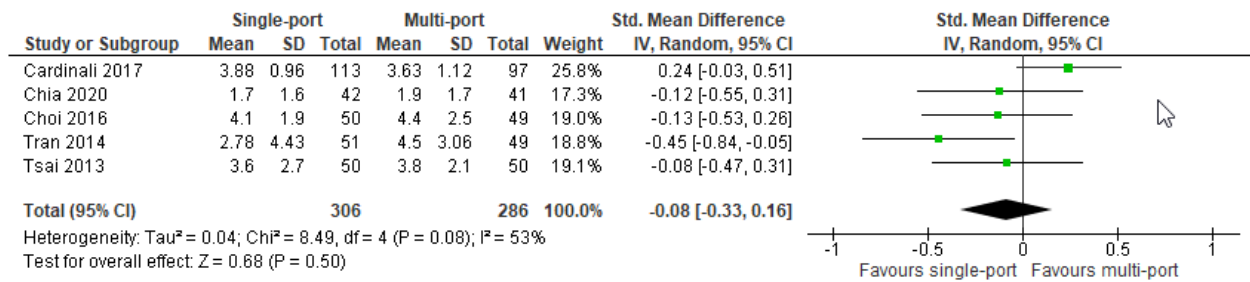

Supplement: Supplementary file 3 [file medi-101-e30820-s003.pdf]
